# Treatment of Vesical Calculus in Children

**Published:** 1904-07-23

**Authors:** 


### TREATMENT OF VESICAL CALCULUS IN CHILDREN.

Douglas Drew[1] advocates supra-pubic lithotomy in children in preference to lithopaxy because, (1) it is more likely to result in a permanent cure; as recurrence is usually due to a particle of the stone forming the nucleus of a fresh calculus as a result of faulty evacuation; (2) although probably there is less risk in lithopaxy there is very little danger in supra-pubic lithotomy, especially in children where the bladder is still an abdominal organ; (3) children often suffer much after lithopaxy from irritation of the bladder, pain on micturition, and the passage of small fragments of stone, and in some cases another anæsthetic may be necessary; whereas after supra-pubic lithotomy the result is certain, and the child will have no further trouble. The wound will probably heal by first intention, and the patient be well in a fortnight.

[1] Brit. Jour. Chil. Dis., July 1904.

